# Innovations in vital signs measurement for the detection of hypertension and shock in pregnancy

**DOI:** 10.1186/s12978-018-0533-4

**Published:** 2018-06-22

**Authors:** Nicola Vousden, Hannah L. Nathan, Andrew H. Shennan

**Affiliations:** Department of Women and Children’s Health, School of Life Course Sciences, King’s College London, London, UK

**Keywords:** Pregnancy, Low resource, Vital signs, Hypertension, Shock index

## Abstract

Approximately 820 women die in pregnancy and childbirth every day worldwide, with 99% of these occurring in low-resource settings. The most common causes of maternal mortality are haemorrhage, sepsis and hypertensive disorders. There are established, effective solutions to these complications, however challenges remain in identifying who is at greatest risk and ensuring that interventions are delivered early when they have the greatest potential to benefit. Measuring vital signs is the first step in identifying women at risk. Overstretched or poorly trained staff and inadequate access to accurate, reliable equipment to measure vital signs can potentially result in delayed treatment initiation. Early warning systems may help alert users to identify patients at risk, especially where novel technologies can improve usability by automating calculations and alerting users to abnormalities. This may be of greatest benefit in under-resourced settings where task-sharing is common and early identification of complications can allow for prioritisation of life-saving interventions. This paper highlights the challenges of accurate vital sign measurement in pregnancy and identifies innovations which may improve detection of pregnancy complications.

## Background

Haemorrhage is the leading cause of maternal mortality worldwide (27.1%), followed by hypertensive disorders (14.0%) and sepsis (10.7%) [1]. The individual treatment components of antibiotics for sepsis, uterotonics and blood transfusion for haemorrhage and magnesium sulfate for eclampsia have proven efficacy several decades ago and are readily available in most settings [2]. Despite this and recent efforts to reduce maternal mortality, in 2015, an estimated 820 women died every day as a result of pregnancy and childbirth, 99% of which occurred in low-resource settings [3].

Timely identification and treatment of pregnancy complications is critical in order to save lives. Yet overstretched and inadequately trained staff and insufficient access to reliable, accurate equipment can cause delays in diagnosis and management of maternal complications, thus contributing to maternal mortality and serious morbidity [4]. The need for early recognition of abnormal vital signs has been highlighted by the Confidential Enquiries report. It has been identified that failure to recognize and act on deteriorating vital signs in a timely manner contributed to avoidable maternal deaths [5, 6]. Strategies to improve timely recognition of women who require intervention to treat life-threatening pregnancy complications may prevent morbidity and mortality. Especially in low-resource settings, where the burden of disease from preventable conditions is so great and accessing appropriate care is critical [4].

This commentary aims to highlight the challenges of accurate vital sign measurement in pregnancy and describe recent innovations that may aid in earlier detection of complications and reduction of maternal morbidity worldwide.

### Detecting hypertension in pregnancy

Approximately, 10% of women experience hypertension (> 140/90) during pregnancy [7]. Pre-eclampsia, new hypertension with proteinuria or end-organ damage after 20 weeks of gestation [8] can manifest in the absence of physical symptoms. Despite promising advances (biomarkers, Doppler velocimetry and prediction models [9]), there are currently no specific screening tests for pre-eclampsia that have sufficient clinical and cost effectiveness to be uniformly adopted into clinical practice [10]. Blood pressure (BP) monitoring therefore remains the most important and frequent screening test undertaken during the antenatal period [11]. It is essential for detecting and monitoring pre-eclampsia, enabling antihypertensive and prophylactic anticonvulsant therapy and appropriate transfer to higher care facilities for timed delivery. Indeed, failure to accurately measure, understand and act on abnormal blood pressures in pregnancy results in increased mortality and morbidity [12, 13].

There are several methods of blood pressure measurement. Auscultation using a sphygmomanometer is a relatively low-cost technique, but this requires a skilled observer to accurately auscultate to the nearest 2 mmHg. Previous studies have demonstrated that this infrequently occurs and that 78% of readings obtained by clinicians in antenatal clinic end in a zero [14]. This observer preference is a source of error in measuring vital signs. Additionally, mercury devices are no longer in clinical use in Europe due to concerns over the toxicity of mercury. Aneroid devices replace the mercury column but still rely on accurate auscultation. They also require more regular maintenance and calibration than mercury, with studies demonstrating that 53% of aneroid devices in the UK General Practice read an error of more than +/− 3 mmHg, which is significantly more than mercury or automated devices (8%) [15].

Automated BP devices use oscillometry to avoid the necessity of auscultation by a skilled user. It is recommended by the British Society of Hypertension and the European Society of Hypertension that they are independently validated to ensure accuracy [16]. However, there are hundreds of commercially available automated devices and only a few have been successfully validated. Even fewer have been validated as accurate in the pregnant population [17]. Automated devices are prone to underestimate BP in women with pre-eclampsia, and this can result in missing a diagnosis of hypertension in women at high-risk of pregnancy complications [18].

Self-monitoring in pregnancy is increasing in popularity, and evidence suggests that it is acceptable to women [9]. However, accuracy is dependent on use of a device validated for home use, of which only five have undergone testing using accepted protocols [10]. Evidence supporting the safety and effectiveness of self-monitoring in improving clinical outcomes is scarce and therefore current guidance on frequency of monitoring is lacking [10]. Wearable technologies have become a recent priority in several global heath funding calls. Wireless vital sign measurements may be of benefit during acute hospital admissions and labour, but current evidence to support their use is limited [19].

### Detecting shock in pregnancy

Given that the majority of maternal deaths are a result of haemorrhage or sepsis it is crucial to measure vital signs in order to allow for early detection of shock. Shock is defined as inadequate tissue perfusion and is classically defined by the presence of tachycardia, hypotension and eventually poor end-organ perfusion [20]. Detecting shock in pregnancy can be complicated by the physiological changes of pregnancy including increased circulatory blood volume. This means that a large volume of blood can be lost before clinical decompensation [20]. Therefore, physiological parameters of shock used outside of pregnancy may not be directly comparable during pregnancy.

Sepsis is defined as life-threatening organ dysfunction caused by a dysregulated host response to infection [21]. However, international guidelines and criteria of sepsis are not specific for pregnancy and some vital sign thresholds may not be appropriate. For example, women experience a physiological baseline tachycardia in pregnancy that may render the threshold of heart rate (HR) > 90 bpm too low. This may be especially problematic in low-resource countries with high rates of anaemia in the pregnant population. In 2017, the WHO defined maternal sepsis as a life-threatening condition defined by organ dysfunction resulting in infection during pregnancy, childbirth, post-abortion or postpartum period [22]. However, this definition does not set thresholds for vital signs that should alert health care providers (HCPs) that the mother is at risk. The UK Sepsis Trust, in collaboration with the National Institute for Health and Care Excellence (NICE), have recently released guidelines and a maternal sepsis tool to improve detection of sepsis in pregnancy. These list ‘amber’ and ‘red’ flag criteria specific to maternal sepsis that should trigger initiation of management such as intravenous fluids and blood cultures [23].

Alongside the measurement of HR and BP to detect shock secondary to haemorrhage or sepsis, Shock Index (SI), is a promising marker of compromise in pregnancy. SI is the ratio of HR to systolic BP. In the non-pregnant population, it was proposed as an earlier marker of blood loss in patients with gastrointestinal haemorrhage over 60 years ago [24]. There have been a number of studies outside of pregnancy that have demonstrated that an elevated SI is associated with adverse outcomes or mortality secondary to haemorrhage [25, 26] or sepsis [27]. The potential for Shock Index as a predictive marker in pregnancy was first explored in women with early pregnancy complications [28, 29]. More recently, a number of small case-control studies have explored the use of SI in obstetric haemorrhage [30, 31]. SI has been shown to be significantly higher in those with post-partum haemorrhage (PPH), those receiving transfusion and those requiring hysterectomy compared to controls with a normal blood loss at delivery [32]. Additionally, SI is significantly higher in those that require massive blood transfusion following PPH compared to those requiring less than 10 units of blood [31].

Shock Index may therefore be a useful measure of early compromise following delivery. However, it is vital that the distribution of values in the normal population are understood so that thresholds of abnormality that should alert HCPs can be identified. A retrospective cohort study of 192 low-risk women demonstrated that the mean SI for all pregnancy lengths beyond 12 weeks was 0.79 (+/− 0.13) [33]. This reflected a trend towards lower values in the first trimester, slight increases in the second trimester and decreasing values from 37 weeks onwards with a normal distribution throughout the population. In the first hour after birth, it has been shown that women with a normal blood loss (< 500 ml) have a median SI of 0.66 (0.52–0.89) [34]. The upper limit of 0.89 supports the growing body of literature suggesting that a SI > 0.9 is associated with increased risk of adverse outcomes in pregnancy. For example, a study of 233 women with major PPH ≥ 1500 ml in a UK tertiary setting that a SI ≥ 0.9 had a 100% sensitivity (95% CI 73.5–100) and 43% specificity (95% CI 36.8–50.3) for predicting intensive care unit admission. In comparison, a SI ≥ 1.7 indicated urgent attention was required, with a 25% sensitivity (95% CI 5.5–57.2) and 98% specificity (CI 94.8–99.3) for predicting ICU admission [35]. This was also reflected in the largest study to date, where in 958 women with PPH (≥ 750 ml in Egypt and Nigeria or ≥ 500 ml in Zambia or Zimbabwe) SI was the most consistent predictor of adverse maternal outcomes including maternal death [36].

### Early detection of compromise in pregnancy

Early recognition of abnormal vital signs, including heart rate (HR), blood pressure (BP), respiratory rate, oxygen saturation and temperature can allow for timely identification of clinical deterioration. In high-income settings, it is common for vital signs to be recorded on a paper-based Early Warning System (EWS), a tool to allow for tracking of vital signs according to thresholds of physiological parameters. When abnormal vital signs are identified and plotted, the colour of the paper (normally yellow or red) highlights the severity of deviation from normal, and therefore the need to escalate care [37]. Since the Confidential Enquiries report recommendation in 2003–2005, EWS have been incorporated into maternity care across the UK [38]. Several studies demonstrate that EWS predict morbidity [39, 40] yet there is a lack of evidence demonstrating the clinical effectiveness. It has been reported that EWS improves communication between HCPs and enabled legitimacy for escalation of care, despite significant cultural boundaries and hierarchies that delay appropriate care [41]. However, it is recognised that effectiveness of EWS is dependent on accurate measurement of vital signs, accurate documentation and effective communication [37]. Staffing pressures are perceived as the greatest barriers to their use [38], and this is likely to be of even greater significance in overwhelmed, low-resource environments.

Novel technologies that incorporate EWS calculations and alert HCPs to abnormalities may reduce errors in paper-based recording and delays in communication, potentially saving maternal lives. For example, VitalPAC is a software system that automatically analyses and alerts HCPs to changes in vital signs. Studies have demonstrated that its use improves accuracy of documentation [42, 43] and non-maternity clinical outcomes [44] but the observational, uncontrolled design of these studies limits interpretation of findings. Additionally, it requires transcription of vital signs into the system which may be associated with error and requires technologies that are not widely available in low-resource settings.

A simpler solution is the Microlife CRADLE Vital Sign Alert (VSA), a semi-automated hand-held upper arm device that measures HR, BP and automatically calculates SI. It has been validated as accurate outside of pregnancy [22] as well as in pregnancy, including women with hypertension [45] or hypotension [46]. It has been developed specifically for use in low-resource settings, with low power requirements and a built-in, rechargeable battery that can be charged with a micro-USB (the same as most international phone chargers). Results are shown on a digital display as well as an EWS traffic light. The lights are triggered by both hypertension and SI. A BP of ≥140 or ≥90 will trigger a yellow light with an arrow pointing up to indicate mild hypertension and ≥160 or ≥ 90 will trigger a red light with an arrow pointing up to indicate severe hypertension. Prospective evaluation of these thresholds in 1547 women with pre-eclampsia in South Africa demonstrated that a red or yellow light on admission was associated with significantly increased rates of kidney injury (OR 1.74, CI 1.31–2.33) and the need for magnesium sulfate (OR 3.40, CI 2.24–5.18) and high dependency admission (OR 1.50, CI 1.18–1.91) [47].

Additionally, a SI of < 0.9 will trigger a green light to reassure that the vital signs are within the parameters of normal. A SI of ≥0.9–1.69 will trigger a yellow light with an arrow pointing down to alert the HCP to the potential need for action. A red light with a down arrow is triggered by a SI ≥1.7, highlighting to any cadre of HCP, even those without formal clinical training, that there is a high risk of adverse outcome and immediate action is required. Prospective validation of these thresholds in sepsis and haemorrhage in the pregnant population has recently completed with initial analysis showing promising results. Qualitative data (155 interviews with HCPs and 41 with pregnant women and their families) have indicated that HCPs found the CRADLE VSA easy to use and that the traffic light EWS improved confidence in decision-making and professionalism [48]. Women and their families reported that the traffic light EWS improved understanding of the importance of vital signs in pregnancy [48]. Results of a stepped-wedge randomized controlled trial introducing this device into routine maternity care in 10 low-resource settings with the aim of reducing maternal mortality and morbidity are eagerly anticipated (ISRCTN41244132).

## Conclusion

In conclusion, despite dramatic progress in the last decade, addressing maternal mortality remains appropriately high on the international agenda. Vital signs measurement remains a vital first step in detecting pregnancy abnormalities in order to initiate timely treatments that can save lives. Inadequate access to accurate, reliable equipment in combination with strain on trained health care providers can lead to delay in recognition of pregnancy complications. EWS may be of benefit in alerting HCPs to abnormal results. Novel technologies such as traffic light alerts may present a user-friendly solution. This may be of greatest benefit in under-resourced settings where task-sharing with HCPs with less formal training is common [49].

## Abbreviations

BP: Blood pressure
Bpm: Beats per minute
EWS: Early warning system
HCPs: Health care providers
HR: Heart rate
NICE: National Institute for Health and Care Excellence
PPH: Post-partum haemorrhage
SI: Shock Index
UK: United Kingdom
VSA: Vital sign alert

## References

[1] Say L, Chou D, Gemmill A, Tuncalp O, Moller AB, Daniels J (2014). Global causes of maternal death: a WHO systematic analysis. Lancet Glob Health.

[2] Adam T, Lim SS, Mehta S, Bhutta ZA, Fogstad H, Mathai M (2005). Cost effectiveness analysis of strategies for maternal and neonatal health in developing countries. BMJ.

[3] World Health Organisation (2015). Trends in maternal mortality: 1990 to 2013. Estimates by WHO, UNICEF, UNFPA, the World Bank and the United Nations population division.

[4] Thaddeus S, Maine D (1994). Too far to walk: maternal mortality in context. Soc Sci Med.

[5] Lewis G. The Confidential Enquiry into Materna and Child Health (CEMACH). Saving Mothers Live’s: reviewing maternal deaths to make motherhood safer - 2003-2005. The Seventh Report on Confidential Enquiries into Maternal Deaths in the United Kingdom. London; 2007. http://www.publichealth.hscni.net/sites/default/files/Saving%20Mothers%27%20Lives%202003-05%20.pdf. Accessed 14 May 2018.

[6] Knight MNM, Tuffnell D, Shakespeare J, Kenyon S, Kurinczuk JJ (Eds.) on behalf of MBRRACE-UK. Saving Lives, Improving Mother’s Care- Lessons learned to inform maternity care from the UK and Ireland Confidential Enquiries into Maternal Deaths and Morbidity 2013–15;2017.

[7] Duley L (2009). The global impact of pre-eclampsia and eclampsia. Semin Perinatol.

[8] Tranquilli AL, Dekker G, Magee L, Roberts J, Sibai BM, Steyn W (2014). The classification, diagnosis and management of the hypertensive disorders of pregnancy: a revised statement from the ISSHP. Pregnancy Hypertens.

[9] Duhig KE, Shennan AH (2015). Recent advances in the diagnosis and management of pre-eclampsia. F1000Prime Rep.

[10] Hodgkinson JA, Tucker KL, Crawford C, Greenfield SM, Heneghan C, Hinton L, et al. Is self monitoring of blood pressure in pregnancy safe and effective? BMJ. 2014;18(349):g6616. 10.1136/bmj.g6616 25406132

[11] Nathan HL, Duhig K, Hezelgrave NL, Chappell LC, Shennan AH (2015). Blood pressure measurement in pregnancy. Obstet Gynaecol..

[12] Cantwell R, Clutton-Brock T, Cooper G, Dawson A, Drife J, Garrod D (2011). Saving Mothers’ lives: reviewing maternal deaths to make motherhood safer: 2006-2008. The eighth report of the confidential enquiries into maternal deaths in the United Kingdom. BJOG.

[13] McCaw-Binns AM, Ashley DE, Knight LP, MacGillivray I, Golding J (2004). Strategies to prevent eclampsia in a developing country: I. Reorganization of maternity services. Int J Gynaecol Obstet.

[14] Wen SW, Kramer MS, Hoey J, Hanley JA, Usher RH (1993). Terminal digit preference, random error, and bias in routine clinical measurement of blood pressure. J Clin Epidemiol.

[15] Coleman AJ, Steel SD, Ashworth M, Vowler SL, Shennan A (2005). Accuracy of the pressure scale of sphygmomanometers in clinical use within primary care. Blood Press Monit.

[16] O’Brien E, Atkins N, Stergiou G, Karpettas N, Parati G, Asmar R (2010). European Society of Hypertension International Protocol revision 2010 for the validation of blood pressure measuring devices in adults. Blood Press Monit.

[17] Natarajan P, Shennan AH, Penny J, Halligan AW, de Swiet M, Anthony J (1999). Comparison of auscultatory and oscillometric automated blood pressure monitors in the setting of preeclampsia. Am J Obstet Gynecol.

[18] Reinders A, Cuckson AC, Lee JT, Shennan AH (2005). An accurate automated blood pressure device for use in pregnancy and pre-eclampsia: the microlife 3BTO-A. BJOG.

[19] Boatin AA, Wylie BJ, Goldfarb I, Azevedo R, Pittel E, Ng C (2016). Wireless vital sign monitoring in pregnant women: a functionality and acceptability study. Telemed J E Health.

[20] Plaat F (2008). Anaesthetic issues related to postpartum haemorrhage (excluding antishock garments). Best Pract Res Clin Obstet Gynaecol.

[21] Singer M, Deutschman CS, Seymour CW, Shankar-Hari M, Annane D, Bauer M (2016). The third international consensus definitions for sepsis and septic shock (Sepsis-3). JAMA.

[22] de Greeff A, Nathan H, Stafford N, Liu B, Shennan AH (2008). Development of an accurate oscillometric blood pressure device for low resource settings. Blood Press Monit.

[23] NICE (2016). NICE clinical guideline 51 Sepsis: recognition, diagnosis and early management: 1–50.

[24] Allgower M, Burri C (1967). Shock index. Deutsche Med Wochenschr (1946).

[25] Cannon CM, Braxton CC, Kling-Smith M, Mahnken JD, Carlton E, Moncure M (2009). Utility of the shock index in predicting mortality in traumatically injured patients. J Trauma.

[26] Sloan EP, Koenigsberg M, Clark JM, Weir WB, Philbin N (2014). Shock index and prediction of traumatic hemorrhagic shock 28-day mortality: data from the DCLHb resuscitation clinical trials. West J Emerg Med.

[27] Yussof SJ, Zakaria MI, Mohamed FL, Bujang MA, Lakshmanan S, Asaari AH (2012). Value of shock index in prognosticating the short-term outcome of death for patients presenting with severe sepsis and septic shock in the emergency department. Med J Malaysia.

[28] Birkhahn RH, Gaeta TJ, Bei R, Bove JJ (2002). Shock index in the first trimester of pregnancy and its relationship to ruptured ectopic pregnancy. Acad Emerg Med.

[29] Birkhahn RH, Gaeta TJ, Van Deusen SK, Tloczkowski J (2003). The ability of traditional vital signs and shock index to identify ruptured ectopic pregnancy. Am J Obstet Gynecol.

[30] Le Bas A, Chandraharan E, Addei A, Arulkumaran S (2014). Use of the “obstetric shock index” as an adjunct in identifying significant blood loss in patients with massive postpartum hemorrhage. Int J Gynecol Obstet.

[31] Sohn CH, Kim WY, Kim SR, Seo DW, Ryoo SM, Lee YS (2013). An increase in initial shock index is associated with the requirement for massive transfusion in emergency department patients with primary postpartum hemorrhage. Shock.

[32] Eppes CS, Schupp J, Dildy G. 272: shock index: a potential criterion for a maternal early warning system. Am J Obstet Gynecol. 214(1):S159.

[33] Borovac-Pinheiro A, Pacagnella RC, Morais SS, Cecatti JG (2016). Standard reference values for the shock index during pregnancy. Int J Gynecol Obstet.

[34] Nathan HL, Cottam K, Hezelgrave NL, Seed PT, Briley A, Bewley S (2016). Determination of normal ranges of shock index and other haemodynamic variables in the immediate postpartum period: a cohort study. PLoS One.

[35] Nathan HL, El Ayadi A, Hezelgrave NL, Seed P, Butrick E, Miller S (2015). Shock index: an effective predictor of outcome in postpartum haemorrhage?. BJOG.

[36] El Ayadi AM, Nathan HL, Seed PT, Butrick EA, Hezelgrave NL, Shennan AH (2016). Vital sign prediction of adverse maternal outcomes in women with hypovolemic shock: the role of shock index. PLoS One.

[37] Jones M (2012). NEWSDIG: the National Early Warning Score Development and implementation group. Clin Med (Lond).

[38] Isaacs RA, Wee MY, Bick DE, Beake S, Sheppard ZA, Thomas S (2014). A national survey of obstetric early warning systems in the United Kingdom: five years on. Anaesthesia.

[39] Carle C, Alexander P, Columb M, Johal J (2013). Design and internal validation of an obstetric early warning score: secondary analysis of the intensive care National Audit and research Centre case mix Programme database. Anaesthesia.

[40] Singh S, McGlennan A, England A, Simons R (2012). A validation study of the CEMACH recommended modified early obstetric warning system (MEOWS). Anaesthesia.

[41] Mackintosh N, Watson K, Rance S, Sandall J (2014). Value of a modified early obstetric warning system (MEOWS) in managing maternal complications in the peripartum period: an ethnographic study. BMJ Qual Saf.

[42] Pullinger R, Wilson S, Way R, Santos M, Wong D, Clifton D (2017). Implementing an electronic observation and early warning score chart in the emergency department: a feasibility study. Eur J Emerg Med.

[43] Schmidt PE, Meredith P, Prytherch DR, Watson D, Watson V, Killen RM (2015). Impact of introducing an electronic physiological surveillance system on hospital mortality. BMJ Qual Saf.

[44] Mitchell C, Meredith P, Richardson M, Greengross P, Smith GB (2016). Reducing the number and impact of outbreaks of nosocomial viral gastroenteritis: time-series analysis of a multidimensional quality improvement initiative. BMJ Qual Saf.

[45] Nathan HL, de Greeff A, Hezelgrave NL, Chappell LC, Shennan AH (2015). An accurate semiautomated oscillometric blood pressure device for use in pregnancy (including pre-eclampsia) in a low-income and middle-income country population: the microlife 3AS1-2. Blood Press Monit..

[46] Nathan HL, de Greeff A, Hezelgrave NL, Chappell LC, Shennan AH (2015). Accuracy validation of the microlife 3AS1-2 blood pressure device in a pregnant population with low blood pressure. Blood Press Monit.

[47] Nathan HL, Seed PT, Hezelgrave NL, De Greeff A, Lawley E, Anthony J, et al. Early warning system hypertension thresholds to predict adverse outcomes in pre-eclampsia: A prospective cohort study. Pregnancy Hypertens. 2017. 10.1016/j.preghy.2017.11.003 PMC6008490 29175171

[48] Nathan HL, Boene H, Munguambe K, Sevene E, Akeju D, Adetoro OO (2018). The CRADLE vital signs alert: qualitative evaluation of a novel device designed for use in pregnancy by healthcare workers in low-resource settings. Reprod Health.

[49] Dawson AJ, Buchan J, Duffield C, Homer CS, Wijewardena K (2014). Task shifting and sharing in maternal and reproductive health in low-income countries: a narrative synthesis of current evidence. Health Policy Plan.

